# Genetic Screening for Growth Hormone Therapy in Children Small for Gestational Age: So Much to Consider, Still Much to Discover

**DOI:** 10.3389/fendo.2021.671361

**Published:** 2021-05-28

**Authors:** Claudio Giacomozzi

**Affiliations:** U.O.C. of Pediatrics, Department of Maternal and Child Health, Carlo Poma Hospital, ASST-Mantova, Mantova, Italy

**Keywords:** small for gestational age, genetics, growth hormone, screening, short stature children, SGA

## Abstract

Children born small for gestational age (SGA), and failing to catch-up growth in their early years, are a heterogeneous group, comprising both known and undefined congenital disorders. Care for these children must encompass specific approaches to ensure optimal growth. The use of recombinant human growth hormone (rhGH) is an established therapy, which improves adult height in a proportion of these children, but not with uniform magnitude and not in all of them. This situation is complicated as the underlying cause of growth failure is often diagnosed during or even after rhGH treatment discontinuation with unknown consequences on adult height and long-term safety. This review focuses on the current evidence supporting potential benefits from early genetic screening in short SGA children. The pivotal role that a Next Generation Sequencing panel might play in helping diagnosis and discriminating good responders to rhGH from poor responders is discussed. Information stemming from genetic screening might allow the tailoring of therapy, as well as improving specific follow-up and management of family expectations, especially for those children with increased long-term risks. Finally, the role of national registries in collecting data from the genetic screening and clinical follow-up is considered.

## Introduction

Growth failure is a frequent reason for referral to a pediatric endocrinologist, and short stature in children born small for gestational age (SGA) is among the indications for the use of recombinant human growth hormone (rhGH).

Children with birth weight and/or length two standard deviations (SDs) below the mean, compared with reference standards for gestational age and gender, are defined as SGA. SGA children may or may not have a history of intra-uterine growth retardation (IUGR). SGA patients, as well as those with IUGR, have been extensively investigated and several conditions have been associated with fetal growth restrictions (1), which are classified into three main categories: maternal, placental and fetal. Maternal causes, including acquired disease (infections, chronic inflammatory disease, anemia, depression) or unhealthy lifestyle (smoking, alcohol and/or drug abuse), represent a minor percentage of causes. Maternal and placental causes are often diagnosed by ultrasound and an in-depth clinical follow-up, enabling the medical management to be tailored and accurate. In cases where gestational follow-up does not reveal any potential association, growth restriction is more likely to be related to intrinsic fetal disorders. Ultimately, if gestational follow-up is missing or inappropriate, it may be difficult to establish the cause of the growth failure.

About 90% of SGA newborns show catch-up growth in the first 2 years of life, their height normalizing by 4 years of age (2). However, about 10% of SGA children remain permanently short, and achieve an adult height less than -2 SDs (3, 4).

## Recombinant Human Growth Hormone

In 2001, rhGH was approved for use in short SGA children, older than 2 years in the USA and older than 4 years in Europe, based on trials demonstrating increased height velocity and improved height gain in children born SGA compared with untreated children. The effects of rhGH administration in the short-term were evaluated by a meta-analysis including 4 independent, randomized, controlled, multicenter studies conducted over 2 years in short pre-pubertal children born SGA (5). Treated SGA children showed a dose-dependent increase in growth, with a significantly accelerating growth rate compared with the untreated group, and an improved final height (5). The first report from a long-term trial showed that a high proportion of short SGA children achieved an adult height (AH) within the target height range, without impact on the onset, progression and duration of puberty, or pubertal height gain (6).

Short SGA adolescents starting rhGH therapy at an early pubertal stage have modest and variable height gain. An AH > –2.5 SDs can be expected in only one third of patients, especially in those with a smaller height deficit at onset of rhGH treatment (7). Some authors suggest that doubling the rhGH dose, particularly during puberty (GH 2 mg/m^2^ per day) may improve height gain and adult height, and that the combined GH/gonadotropin-releasing hormone a treatment at the onset of puberty, may be a successful strategy to improve short stature in SGA adolescents with a height <140 cm at the onset of puberty (8, 9). However, the small number of treated subjects and the lack of further controlled randomized trials leave this issue inconclusive.

An epi-analysis of the first AH data from SGA children enrolled in four randomized trials comparing the growth-promoting efficacy of two continuous rhGH regimens (0.033 or 0.067 mg/kg per day for ~10 years, starting at ~5 years of age) (10), and an epi-analysis of the AH results published earlier than 2005 (11), confirmed that rhGH therapy is effective and safe in reducing the AH deficit in SGA patients. Contrary to previous evidence (8, 9), long-term higher rhGH doses (0.067 mg/kg per day) did not correlate with a better height gain or AH, than lower doses (0.033 mg/kg per day) (11, 12), even in adolescents (13).

In 2009, a meta-analysis of randomized, controlled trials (four RCTs including 391 children) on the impact of rhGH therapy on AH in short SGA children concluded that AH of the rhGH-treated group significantly exceeded controls by 0.9 SDs (mean height gain 1.5 SDs in treated vs 0.25 SDs in untreated SGA subjects) (14). Once again, no significant difference in AH was observed between different rhGH dose regimens. However, the response to therapy was highly variable, and additional studies were suggested to identify the good responders.

## Markers for Predicting Good Responders

Despite the efforts to identify variables predictive of good response to long-term rhGH therapy and the development of prediction models (15–18), to date, no pre-treatment variables have been found to be reliable for predicting rhGH response. The ability to predict a patient’s response to rhGH would not only quantify the magnitude of height gain to be expected at the end of treatment, but, most importantly, would avoid treating non-responders, especially those who have intrinsic risk factors. The identification of good and non-responders would not only optimize the use of financial and human resources, but also prevent patient frustration and disappointment potentially after years of treatment that does not provide the expected benefit.

Clinical trials identified younger chronological and bone age at the start of treatment with rhGH as major determinants of height gain in SGA children. No other considered parameters correlated significantly with the height gain SDs from the start of rhGH treatment until AH (12). Interestingly, the AH SDs correlated positively with height SDs at the start of rhGH treatment, target height SDs, and pre-treatment height velocity SDs (12). Growth hormone secretion did not correlate with height gain in SGA children considered GH deficient by provocative tests (12). Prediction models, conversely, suggested rhGH dose was among the major predictive markers, along with weight SDs, height velocity SDs, chronological age and mid-parent height SDs (17). Conflicting data and poor applicability of predictive models to daily clinical practice have reduced the impact of these findings (18). Currently, there is no specific clinical feature that identifies a patient as one who will not benefit from rhGH therapy, unless the patient’s phenotype (and genotype) is diagnostic for a disease already known to be unresponsive to rhGH therapy.

The polymorphic deletion in exon 3 of the GH receptor (d3-GHR) has been investigated as a potential predictive marker of growth response in SGA patients, as previously shown in children with other rhGH-treated growth disorders (19). However, though the height velocity increase has been positively correlated with the presence of one or two copies of the d3-GHR allele, this association was observed only in the first and second year of treatment. No correlation was found with overall height gain and AH. In a large randomized controlled study conducted in Spain in SGA children, neither growth velocity nor height gain differed among the d3/fl-GHR genotypes over two years of rhGH therapy (20). Furthermore, the spontaneous growth rate in the untreated group did not correlate with d3/fl-GHR genotype.

Although polymorphisms in *IGF1* and *IGF1R* genes may be potential candidate markers, only one polymorphism in the promoter region of the *IGF1* gene has been associated with growth in SGA children, and its exact contribution in clinical practice remains to be elucidated (21).

Multi-allele single nucleotide polymorphisms associated with insulin sensitivity or insulin secretion have been positively associated with height velocity in the first year of rhGH therapy, but not with growth and IGF-1 responses in the long-term (22).

Overall, these data raise the question of whether the search for predictors of rhGH response among variables already linked with GH deficiency should continue, or if resources would be better spent personalizing the approach and better defining the pathogenesis underlying each individual SGA child.

## Safety

The safety of rhGH therapy is a crucial issue in SGA children, as they represent an extremely heterogeneous and still poorly characterized group of patients.

A twelve-year study on rhGH therapy in SGA children has shown that rhGH therapy is well tolerated (13). Although both higher fasting and glucose-stimulated insulin levels were observed during treatment, 6 months after rhGH discontinuation, blood insulin concentrations normalized. Glycolated hemoglobin levels of SGA patients remained within normal ranges during rhGH treatment, irrespective of the dosage (0.033 versus 0.067 mg/kg/week) (23). Furthermore, rhGH therapy demonstrated a beneficial effect on serum lipid profiles, body composition, bone mineral density, and head growth.

Data on morbidity and mortality were collected during up to 25 years of follow-up in the SAGhE project (24, 25), the largest independent post-marketing surveillance study of rhGH treatment published so far. In the young adult SGA population treated with rhGH therapy during childhood, overall cancer mortality and morbidity were not increased. All-cause mortality was significantly increased when analyzed for all countries involved in the study (eight from across Europe) (SMR 1·5 [1·1–1·9]). However, when analyzed separately, the risk was significantly increased only in France (SMR 1·7 [1·2–2·4]), but not significantly in the other countries combined (SMR 1·2 [0·8–1·9]). The increased mortality risk was associated with diseases of the circulatory system. No significant association between mortality and rhGH dose, either mean daily or cumulative, was found.

Although SAGhE data are reassuring overall about mortality risk associated with rhGH therapy in SGA children, both cancer morbidity and mortality and all-cause mortality were significantly higher in the syndromic group treated with rhGH compared with the general population. These data suggest that some genetic syndromes have intrinsic risk of morbidity and mortality, which might be exacerbated by rhGH therapy. As a proportion of SGA patients harbor genetic abnormalities, the potential risk requires long-term surveillance in those treated with rhGH.

## SGA Short Stature as “Neonatal Idiopathic Short Stature”

Before rhGH approval for SGA, low weight or short length at birth were well known findings in many genetic disorders, including Cornelia De Lange (1933) (26), Silver-Russell syndrome (1953-1954) (27, 28), primary IGF1 signaling anomalies (1996) (29), and more recently Gs-alpha protein-related disorders (2018) (30). Research has established the importance of distinguishing children with these conditions (who do not benefit from rhGH) from those born SGA (who might). Therefore, it seems entirely logical to expect to find more genetic defects misdiagnosed in patients who are currently labeled as SGA. Moreover, the broad individual variability of response to rhGH treatment in the SGA population is mainly due to the heterogeneous pathogenesis underlying fetal and post-natal growth restriction in these children.

Since early 2000, genetic research has resulted in the development of many sophisticated diagnostic techniques to analyze both copy number variations and punctiform mutations in an increasing number of genes simultaneously and faster. Current evidence suggests that a considerable number of SGA patients without catch-up growth carry monogenic, polygenic, or epigenetic variants, which explain their growth failure. Some short SGA patients might be considered to have a sort of neonatal idiopathic short stature (ISS), especially those without an increased risk for metabolic and cardiovascular disease in later life. SGA and ISS share the same challenges for elucidating the genetic/epigenetic cause(s) of these conditions and for finding successful treatment(s). Their similarity is supported by case studies, showing that skeletal dysplasia accounts for more than 20% of short stature in both SGA and ISS children (31). Moreover, *IGF1*, *IGF1R*, and *SHOX* mutations have been demonstrated in both short SGA and ISS children, although in a smaller percentage of cases (32). Both represent a group of patients categorized just on the basis of auxological parameters. In fact, the SGA condition is comprised of a large group of diseases sharing similar growth patterns but with many other different features and implications, both in children and in adults. Genome sequencing techniques have allowed the determination of defects in genes related to growth cartilage in patients with isolated short stature, who could be defined SGA only by their short birth length (33). The genes involved in endochondral ossification or cartilage extracellular matrix synthesis are responsible for very mild forms of bone dysplasia that may be clinically classified as ISS or SGA, according only to the timing of the short stature definition.

The identification of good responders is, therefore, a priority to successfully treat SGA patients, as well as ISS patients.

## Advances of Genetics in SGA

The diagnostic capabilities of medical genetics are constantly evolving, and great strides forward have been made in understanding the genetic profile underlying many SGA children. Extensive research has contributed to this progress, and, in highlighting a few key papers, we acknowledge that the few cited articles are far from an exhaustive list. A representative review of what has been achieved so far, is reported by Peeters et al. (34), who demonstrated that a more advanced approach with accurate genotyping is successful in finding new potential pathways involved in the IUGR/SGA condition. This extensive genetic work-up led to identification of pathogenic variants in *ELAC2*, *CEP57*, and *HNRHPH1* genes by exome sequencing, the potential involvement of chromosomal regions such as 5q.35 and 22q.11 by SNP array analysis, and different patterns of methylation in specific regions regulating the *GNAS* gene (already thought to potentially affect growth) by genome-wide methylation analysis.

It has been reported that about 13% of short SGA patients treated with rhGH and presenting with advanced bone age at some point of their therapy, carried an Aggrecan (*ACAN*) gene mutation (35). SGA patients harboring *ACAN* mutations are characterized by additional features such as midface hypoplasia, joint problems, and broad great toes, and seem to benefit from both rhGH and gonadotropin-releasing hormone analogue to improve their AH, although the small cohort of studied patients does not allow definitive conclusions.

An early genetic approach may help to elucidate the molecular defect underlying the SGA condition and tailor the appropriate treatment, if available. Parents might be informed in advance and more accurately on ‘what it is reasonable to expect’, and this could positively impact on patients’ and families’ compliance and, ultimately, on clinical results.

## Potential Role of Genetic Screening in Short SGA Children

There is increasing evidence that genetic analysis may be used not only as a diagnostic tool but also for safety screening in SGA children.

According to the 2001 international consensus on SGA children (36), apart from Bloom syndrome and a few other chromosomal anomalies (trisomy 18 and 21, monosomy X), genetic investigations have not generally been considered in short SGA children before starting rhGH. However, many other syndromic disorders have been associated with a SGA phenotype, such as Rothmund–Thomson syndrome, Louis-Bar syndrome and Nijmegen breakage syndrome, all of which are associated with an increased risk of malignancies. Consequently, the cause of poor growth remains a ‘mystery’ in the majority of SGA children at the onset of rhGH therapy, even in a minor but extremely important percentage with potential cancer-related disorders.

Since 2001, Next Generation Sequencing (NGS) and Whole Exome Sequencing (WES) have revealed the association of an SGA phenotype with syndromic conditions not previously elucidated or whose heterogeneous phenotype-genotype correlation did not allow a proper diagnosis in a proportion of cases (37–40). It should also be considered that the percentage of diagnosis by exome sequencing for children born SGA with persistent short stature and absence of syndromic features is lower than that for syndromic children (33–38). This may suggest a wider involvement of epigenetic mechanisms in isolated short stature pathogenesis than is actually believed (37).

**Figure 1:**
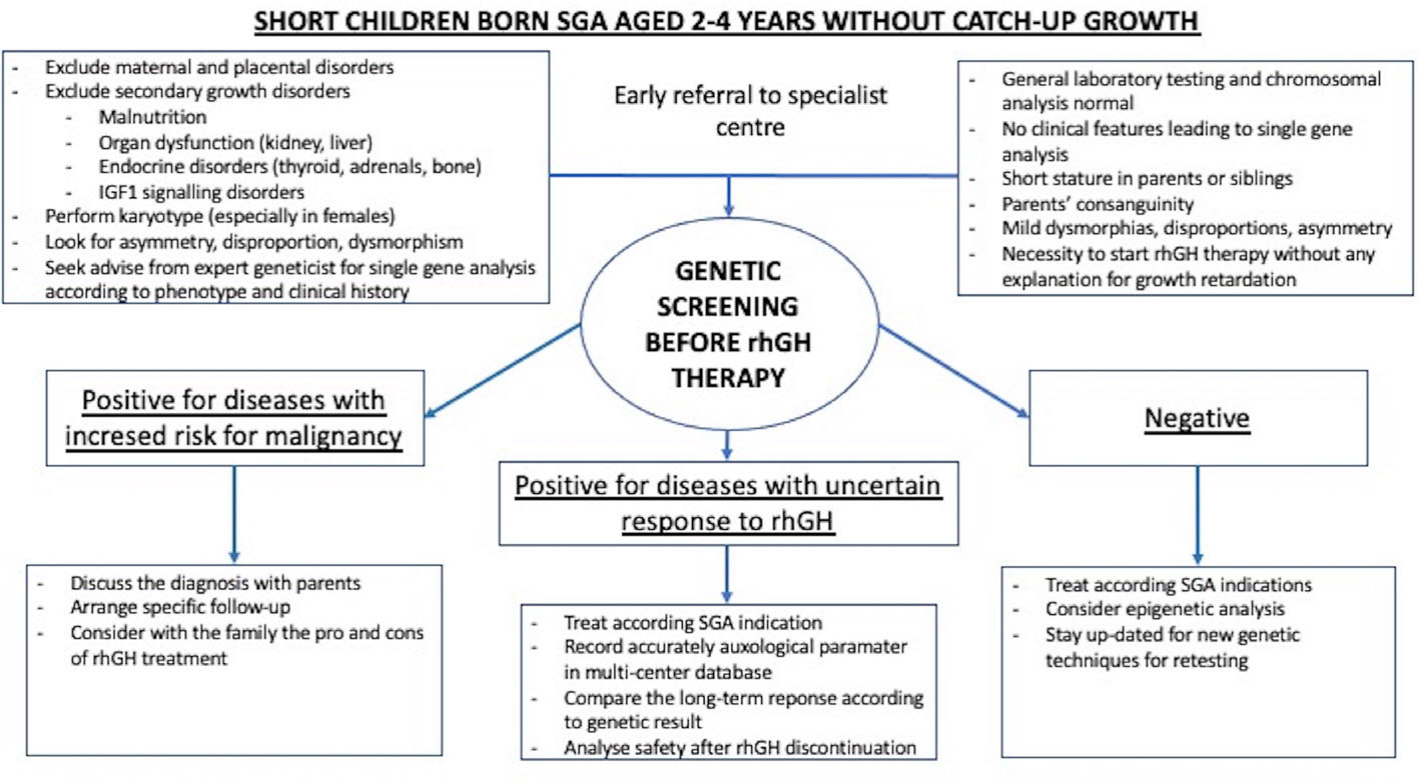
Example of genetic screening strategy included in the work-up and treatment of children born SGA with persistent short stature.

Potentially, genetic screening strategies (**Figure 1**) could increase the safety of rhGH therapy in SGA children undiagnosed at treatment onset. NGS panels, especially set up with known SGA-related genes (example shown in **Table 1**), may work as screening tests, with the advantage over other genetic techniques of a short response time and low cost. In the cases of syndromes with increased risk for malignancy, whose magnitude is largely unknown but might not be negligible in the SGA population, therapy with rhGH should be avoided, or, after extensive discussion with parents and in cases of severe short stature, undertaken only with specific and intensive clinical and laboratory monitoring. Although the real impact of rhGH on cancer risk in many syndromes remains unknown, the most cautious management should be pursued. In case of a ‘negative’ on genetic screening, diagnostic work-up should continue and further investigations should be arranged during treatment to get a definitive diagnosis, and to correlate the response to treatment. Indeed, the magnitude of rhGH efficacy has not been clarified in a significant portion of 'SGA-related genetic diseases’ covered in the theoretical NGS panel in **Table 1**. The genes included in the NGS safety screening panel should be periodically reviewed according to the latest data, to maintain safety at the highest possible level. Naturally, over time, the SGA screening NGS panel might become a diagnostic tool to investigate this population. The use of inclusion criteria for SGA genetic screening should be carefully discussed. On the one hand, selective criteria might improve the percentage of diagnoses (29), but on the other hand, might not be a sufficient safety tool, especially in patients with non-specific phenotypes (38). Among the limits of genetic screening by NGS panels would be gene methylation disorders such as Silver Russel syndrome and Temple syndrome. However, the contribution of these syndromic conditions among SGA with isolated short stature has not been demonstrated significantly so far. For these disorders, specific diagnostic pathways should be arranged before genetic screening according to phenotype, or later for those cases where features are not prognostic and genetic screening has not been helpful. Finally, safety genetic screening may prevent the premature discontinuation of rhGH due to patients’ frustration at unsatisfactory or slow results, thus preventing family disappointment, and waste of public health resources.

**Table 1:** Some genes responsible for pre-natal short stature and included in a genetic screening panel.

	Genes associated with SGA phenotype								Diseases associated with these genes abnormalities
Diseases with increased risk of carcinogenesis	TRIM37	ATR	RBBP8	CENPJ	CEP152	CEP63	NIN	DNA2	Mulibrey nanism, Seckel syndrome, Fanconi anemia, Nijmegen breakage, Rothmund-Thomson syndrome, Louis-Bar syndrome, Neurofibromatosis type I
	ATRIP	RECQL3	FANCA	BRCA1	NBN	RECQL4	ATM	MRE11	
	ANAPC1	NF1							
Disease for which rhGH therapy is generally considered ineffective	FGFR3	IGFALS	IGF1	PIK3R1	IL2RG	GHR	STATSB	PAPPA2	Acondroplasia , Hypocondroplasia, ALS deficiency, IGF l deficiency, SHORT syrdrome, Laron Syrdrome, X-linked severe combined immunodeficiency, GH insensitivity with immunodeficiency, PAPP-A2 deficiency, Pseudohypoparathyroidism
	GNAS1								
Diseases for which it is not yet known whether rhGH is effective or not	SCRAP	CUL7	OBSL1	CCDC8	NIPBL	SMC1A	SMC3	RAD21	Floating-Harbor syndrome, Cornelia de Lange syndrome, 3M syndrome, Meier-Gorlin syndrome, MOPD I, MOPD II, LIG 4 syndrome, XRCC4 syndrome, Severe growth restriction with distinctive facies, Resistance to IGF-1, Multisystem infantile onset autoimmune disease, Short stature with or without advanced bone age and early-onset osteoarthritis Brachydactyly type A, Short stature with nonspecific skeletal abnormalities
	HDAC8	ORC1	ORC4	ORC6	CDT1	CDC6	RNU4ATAC	PCNT	
	LIG4	XRCC4	IGF2	IGF1R	STAT3	ACAN	IHH	NPR2	
Diseases f or which rhGH is approved treatment	SHOX	PTPN11	SOS1	RAF1					Shox deficiency, Noonan Syndrome

The gene panel is based on genes associated with a SGA phenotype and available from current literature (26–28, 35, 37–50). This panel does not include every gene described in the short SGA population at the time of publication, however it aims to provide a realistic perception of the size of SGA population that needs to be further investigated. Genes involved in epigenetic and methylation disorders associated with the SGA phenotype are not included in the panel, as these would not be detected by NGS.

## Potential Role of National Registries

National registries play a fundamental role in collecting data on both efficacy and safety of rhGH during active treatment and long after (51, 52). Unfortunately, the main junction where patients are lost is still the post-discontinuation follow-up, greatly limiting their use and reliability in long-term surveillance programs. Agencies regulating the registries should set up an annual recall system, based on a few but essential parameters to maintain surveillance. Modern telemedicine tools may allow long-term data collection with reduced impact on costs and human resources. Registries should also include the option to enter or adjust the underlying etiology even many years after rhGH discontinuation, to prevent bias related to misdiagnoses. A non-minor issue should be the independence of registries, as most data currently available has been collected by pharmaceutical companies. The sharing of data between different registries would be ideal to reach cohorts of sufficient size to draw conclusive results.

## Conclusion

In conclusion, rhGH is currently the best available therapy to improve adult height in SGA children who do not spontaneously catch-up their growth.

Management of rhGH therapy in SGA children should go beyond prescribing the appropriate dose, IGF1 monitoring and the timing of treatment initiation - it should also include:

- Pre-arranged safety screenings, possibly standardized and internationally recognized;
- Promotion of a continuous diagnostic effort, independently dictated by patient age and duration of rhGH therapy;
- Detailed collection of efficacy and safety data by a network of interconnected registries designed to identify good and bad responders to rhGH therapy.

## Author Contributions

The author confirms being the sole contributor of this work and has approved it for publication.

## Funding

This article has been financially supported by Scientific Seminars International Foundation (Italy), to cover publishing cost through an educational grant received from Merck Healthcare KGaA, Darmstadt, Germany. The sponsor has had no role in data selection or in manuscript preparation.

## Conflict of Interest

CG received honoraria from Novo Nordisk for consultancies as an expert board member; honoraria from Merck Serono as a medical advisor; honoraria from Ferring for article authorship. CG has no non-financial relationships (personal, political, or professional) that may potentially influence the writing of this manuscript.
